# POCUS in the Management of Complications with Barbed Polydioxanone (PDO) Threads in Aesthetic Medicine: Case Report

**DOI:** 10.24908/pocusj.v11i01.19968

**Published:** 2026-04-22

**Authors:** Rhaina Alves Setúbal, Rachel Brazuna Solidonio, Luiz Filipe Barbosa Martins, Daniel Almeida Ferreira Barbosa, Thâmara Manoela Marinho Bezerra, Delane Viana Gondim, Luane Macêdo de Sousa

**Affiliations:** 1 Master's Program, Paulo Picanço School of Dentistry, Fortaleza, CE, Brazil; 2 Postgraduate Program in Dentistry, Federal University of Ceará, Fortaleza, CE, Brazil; 3 Paulo Picanço School of Dentistry, Fortaleza, CE, Brazil

**Keywords:** Case report, Diagnostic imaging, Cosmetic Technique, Polydioxanone, Adverse Effects, POCUS, Point of Care Ultrasound.

## Abstract

Point of care ultrasound (POCUS) is becoming an essential tool in aesthetic medicine, allowing clinicians to evaluate tissues in real time and respond promptly to complications. We present the case of a 42-year-old woman with diabetes who developed swelling, pain, and purulent drainage shortly after undergoing nonsurgical rhinoplasty with hyaluronic acid and polydioxanone (PDO) threads. POCUS quickly revealed an abscess surrounding a thread knot as well as additional fluid collections along the nasal dorsum. These findings guided the surgical plan and enabled real-time visualization during thread removal, improving safety and ensuring complete extraction. Histology later confirmed granulomatous inflammation. The patient recovered without further issues. This case illustrates how POCUS can assist in diagnosing and guiding the treatment of aesthetic complications, reinforcing its value as a simple, non-invasive tool that enhances patient safety and outcomes in everyday clinical practice.

## Introduction

The rising popularity of minimally invasive aesthetic treatments has sparked a surge in the use of barbed polydioxanone (PDO) threads, especially for subtle enhancements like shaping the nose. These threads provide a non-surgical option that comes with benefits such as being absorbable, biocompatible, and generally well tolerated by patients. They come in both smooth and barbed varieties, encouraging collagen production and, in the case of the barbed threads, offering mechanical lifting and gradual skin tightening [1].

While PDO threads are generally considered safe, they can still lead to some adverse effects, which may range from minor issues to more serious complications like infections or tissue damage. In such cases, point of care ultrasound (POCUS) has proven to be an invaluable tool for both diagnosis and intervention. POCUS allows for accurate, real-time localization of threads and precise assessment of surrounding anatomical structures, directly at the patient's bedside. This case report discusses a complication that arose after using PDO threads for nasal tip projection, highlighting the POCUS-assisted removal technique and the histopathological findings, thereby emphasizing the critical role of this imaging modality in enhancing patient safety and optimizing outcomes in aesthetic medicine.

## Case Presentation

The patient was a 42-year-old woman with diabetes. She sought care after undergoing a nasal remodeling procedure that involved the application of hyaluronic acid and the insertion of three PDO threads for nasal support. Three days after the procedure, she began experiencing intense pain, redness, and purulent discharge in the nasal region. Clinical examination confirmed the presence of discharge.

Initial treatment included the application of hyaluronidase, as well as antibiotic therapy with cephalexin (500 mg, every 8 hours for 7 days) and dexamethasone (2 mg, every 6 hours for 2 days). Laser therapy was also performed (Therapy XT, DMC Equipamentos, São Carlos – Brazil), using 3 joules distributed at specific points on the nose, root, dorsum, and tip, with 30 seconds of application at each site. The therapy combined red and infrared wavelengths, aiming to promote healing, reduce inflammation, and drainage.

**Figure 1.**
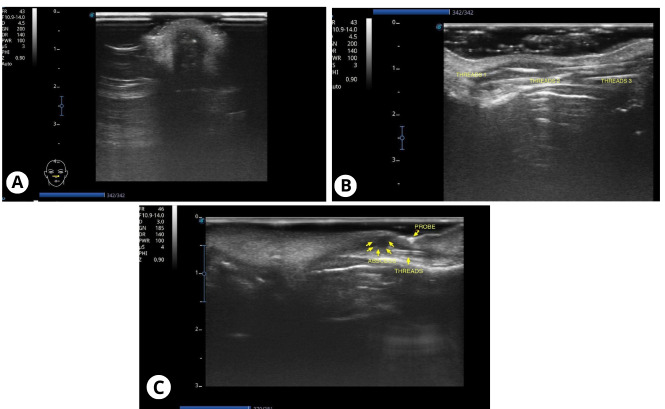
Point of care ultrasound (POCUS) images of the nose. A: A diffuse anechoic area is observed at the tip of the nose, associated with a surrounding hyperechoic region concerning for abscess. B: Three elongated hyperechoic lines are seen along the long axis of the nasal dorsum, inserted in the subcutaneous tissue, consistent with polydioxanone (PDO) threads and surrounded by diffuse anechoic areas (yellow arrows) extending from threads. C: PDO threads in the nose with abscess.

To complement the evaluation, POCUS was used (SAEVO - EVUS 5, Alliage S/A Dental Medical Industries, Ribeirão Preto – Brazil). POCUS showed a poorly defined area, compatible with fluid accumulation (abscess). It involved a dense and highly reflective structure at the center of the lesion, suggesting a PDO thread knot encapsulated by pus (Figure 1A, 1B). Additionally, it was possible to identify the three threads aligned along the nasal dorsum using POCUS, located in the subcutaneous layer and surrounded by hypoechoic areas, reinforcing the diagnosis of an inflammatory process along the thread paths (Figure 1B). This information was essential for surgical planning.

Given the situation, surgical removal of the threads with real-time POCUS guidance was chosen. POCUS allowed for monitoring the introduction of the cannula and the removal of the threads, making the procedure safer and more precise.

**Figure 2.**
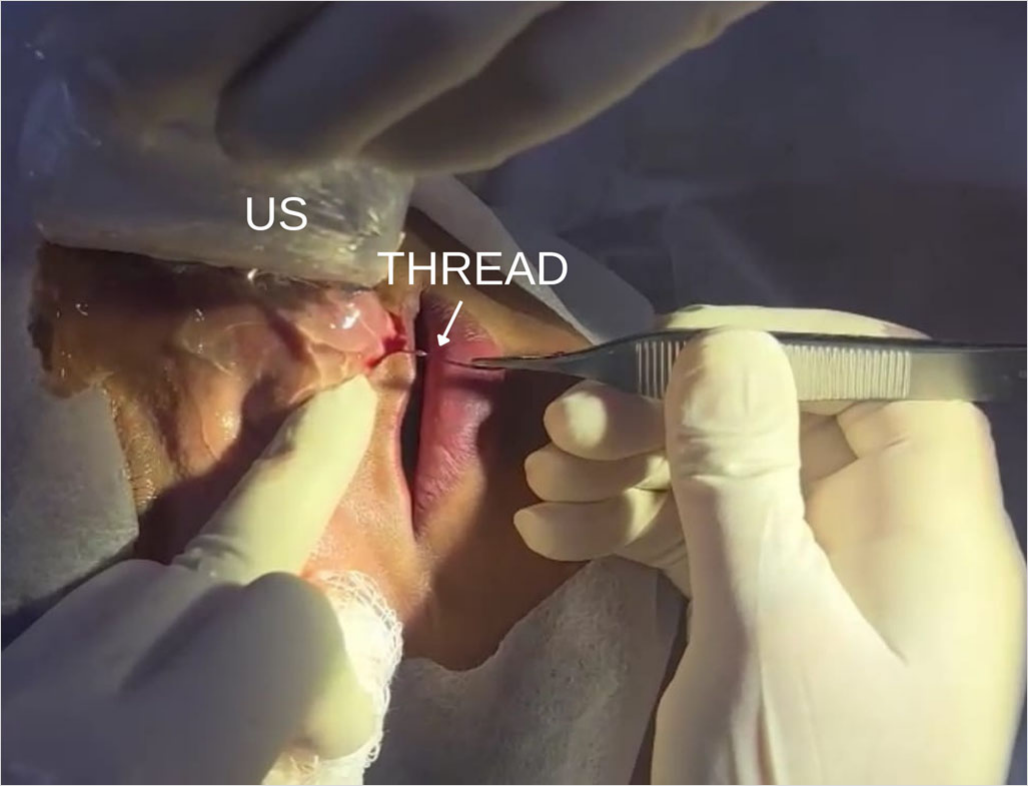
Intraoperative view of nasal incisions with removal of PDO thread using surgical forceps under point of care ultrasound (POCUS) guidance. US, ultrasound.

Anesthesia was performed by blocking the infraorbital foramen with 2% mepivacaine with epinephrine 1:100,000 (DFL Indústria e Comércio S.A, Rio de Janeiro – Brazil), in addition to extraoral local anesthesia with mepivacaine without vasoconstrictor applied to the nasal dorsum. Incisions were made with a No. 15 scalpel blade (Solidor, Itaipava – Brazil) on the columella and nasal dorsum, at points previously identified by POCUS. Through these openings, the PDO threads were visualized and carefully removed with surgical forceps (Golgran, São Caetano – Brazil) (Figure 1C, Figure 2). Continuous POCUS guidance helped to minimize tissue damage and ensure complete removal.

**Figure 3.**
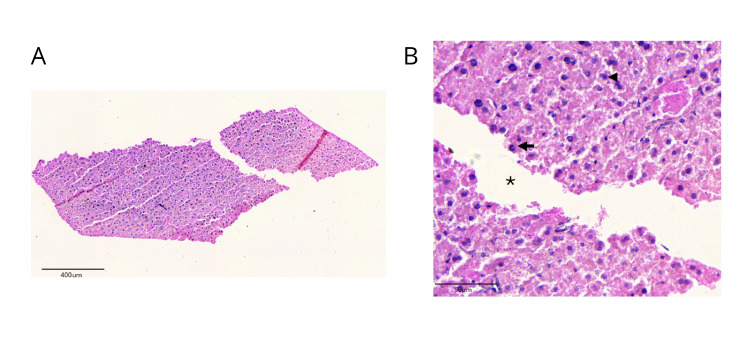
A: Photomicrograph showing the excised granulation tissue surrounding a rectangular empty space corresponding to the transversely cut polydioxanone (PDO) thread. B: Photomicrograph in greater detail showing infiltration of polymorphonuclear neutrophils (arrow), lymphocytes (arrowhead), vascular neoformation and empty space corresponding to the region of the PDO thread (asterisk).

After surgery, the thread surrounded by granulomatous tissue was fixed in 10% formalin (EXODO CIENTÍFICA – Sumaré/SP – Brazil) and processed for a histological slide stained with hematoxylin and eosin (Dinâmica Química Contemporânea LTDA, Indaiatuba/SP – Brazil). This allowed for microscopic analysis of the inflammatory process (Figure 3A, 3B).

## Discussion

The use of barbed PDO threads in rhinoplasty has grown in popularity as a minimally invasive alternative to traditional surgical techniques [1]. These threads are capable of reshaping the nasal contour and stimulating collagen production, providing natural and progressive results with shorter recovery times [1]. Nonetheless, their widespread utilization has been paralleled by an increase in reported complications. The most frequent adverse outcomes include skin wrinkling, extrusion, thread breakage, and, on histology, fibrosis and encapsulation [2,3].

Although less prevalent, local infections represent one of the most critical complications due to their potential to progress to granuloma formation, necrosis, and scarring [4,5]. Surowiak et al. described postoperative bacterial infection related to PDO threads, while Goldan et al. reported cyst formation after thread placement [4,5]. These studies reinforced that although the global incidence of infection is relatively low (2–8.9%), anticipation and early recognition are essential [1,3–6]. This is particularly relevant in high-risk patients, including those with diabetes, who exhibit impaired vascularization and wound healing that may predispose them to more severe outcomes [4,5].

In the present case, clinical findings of purulent drainage were supported by histopathological evidence of necrosis and granulomatous inflammation consistent with a foreign-body reaction, as previously reported [2,6]. Granuloma formation resulting from persistent inflammatory activity has been repeatedly associated with PDO threads and underscores the complexity of management once bacterial contamination occurs.

The most distinctive feature here, however, was the decisive role of POCUS. Previous authors have emphasized that POCUS enables accurate visualization of soft tissues, thread trajectory, and secondary inflammatory changes [7,8]. Mlosek et al. also described how high-frequency ultrasound can characterize nodular and fibrotic complications following thread insertion [9]. In our case, POCUS not only identified an abscess surrounding a central PDO knot, but also delineated fluid collections along the thread pathway, permitting precise mapping of the lesion. During surgery, POCUS provided real-time guidance for cannula introduction and thread removal, enhancing safety and minimizing unnecessary tissue trauma. This practical contribution has also been suggested by Schelke et al., who demonstrated POCUS-guided approaches in the management of filler-related vascular complications [10].

Preventive measures remain a cornerstone in minimizing risks. Bertossi et al. suggested that antibiotic prophylaxis could be considered in patients with systemic comorbidities, although not universally applied [1]. Ryoo et al. further highlighted that improper thread insertion depth increases the likelihood of ischemic complications and subsequent infection [8]. In this case, it is plausible that superficial placement of threads at the nasal tip, compounded by the patient's diabetic status, provided the substrate for early infectious complications.

In summary, this case reinforces that POCUS is a valuable adjunct in aesthetic practice, both for early diagnosis and for guiding intervention. Incorporating POCUS into clinical protocols for minimally invasive procedures offers the potential to improve diagnostic accuracy, reduce complications, and enhance patient safety, especially in high-risk groups such as diabetics.
